# Potential Nutrients from Natural and Synthetic Sources Targeting Inflammaging—A Review of Literature, Clinical Data and Patents

**DOI:** 10.3390/nu13114058

**Published:** 2021-11-13

**Authors:** Sushruta Koppula, Mahbuba Akther, Md Ezazul Haque, Spandana Rajendra Kopalli

**Affiliations:** 1 Department of Integrated Biosciences, College of Biomedical & Health Science, Konkuk University, Chungju 27381, Korea; koppula@kku.ac.kr (S.K.); smritymahbuba@gmail.com (M.A.); 2 Department of Applied Life Science, Graduate School, BK21 Program, Konkuk University, Chungju 27381, Korea; mdezazulhaque@yahoo.com; 3 Department of Bioscience and Biotechnology, Sejong University, Gwangjin-gu, Seoul 05006, Korea

**Keywords:** inflammaging, aging related disorders, low grade inflammation, nutrients, natural herbs, pro-inflammatory cytokines

## Abstract

Inflammaging, the steady development of the inflammatory state over age is an attributable characteristic of aging that potentiates the initiation of pathogenesis in many age-related disorders (ARDs) including neurodegenerative diseases, arthritis, cancer, atherosclerosis, type 2 diabetes, and osteoporosis. Inflammaging is characterized by subclinical chronic, low grade, steady inflammatory states and is considered a crucial underlying cause behind the high mortality and morbidity rate associated with ARDs. Although a coherent set of studies detailed the underlying pathomechanisms of inflammaging, the potential benefits from non-toxic nutrients from natural and synthetic sources in modulating or delaying inflammaging processes was not discussed. In this review, the available literature and recent updates of natural and synthetic nutrients that help in controlling inflammaging process was explored. Also, we discussed the clinical trial reports and patent claims on potential nutrients demonstrating therapeutic benefits in controlling inflammaging and inflammation-associated ARDs.

## 1. Introduction

Aging is a complex physiological and psychological process throughout life where changes occur in many different aspects. Interaction between environmental and genetic factors is the main accelerating force behind aging [1,2]. During the past several decades, research in geroscience has produced several highly interconnected hallmarks for aging progressiveness, namely epigenetics, inflammation, cellular senescence, stem cell exhaustion, mitochondrial dysfunctions, proteostasis, metabolism derangement, and altered intracellular communications [3,4].

As a part of the immune system, inflammation is essential to survive and eliminate the invasion of harmful pathogens, however, this can be detrimental in elderly persons. An array of evidence suggests that an increase in systemic inflammation is a very common phenomenon during aging [5,6]. Inflammaging, first coined by Franceschi et al., is best defined by low-grade, systemic, chronic, asymptomatic, and persistent inflammation [2]. Even though inflammaging is low-grade inflammation, its uncontrolled phenomenon might be a significant risk factor in human aging contributing to their morbidity and mortality [7].

When it comes to elderly, the inflammaging-associated cytokine levels are notably increased, although they remain within the range. The anti-inflammatory response of the body following inflammation is exactly the opposite and controls the inflammatory mechanism. The balance between pro-inflammation and anti-inflammation is essential for the outmost result of inflammation; a similar concept is observed for inflammaging and anti-inflammaging processes. The concept of inflammaging is defined based on the chronic and progressive increase in the pro-inflammatory status and reduction in the ability to respond to different stressors [1].

In general, inflammaging inhibits the ability to fight against infection and wound healing by the impairment in response against new antigens. With progressive aging, a strong correlation between inflammaging and production of proinflammatory cytokines such as tumor necrosis factor-alpha (TNF-α), interleukin (IL)-6, IL-1, and C-reactive protein (CRP) has been established [8,9,10]. An increase in these proinflammatory cytokines can potentiate the loss of muscle strength, bone metabolism, and nutritional status, which can also lead to age-related disorders (ARDs) such as neurodegenerative diseases, arthritis, cancer, atherosclerosis, type 2 diabetes (T2D), cardiovascular disorders (CVDs), and osteoporosis [11,12,13,14].

The inflammaging theory, its pathological changes, and their role in the development of disease, mechanisms, and interventions have been extensively reviewed [15,16]. However, knowledge regarding inflammaging is still incomplete as the cause and risk factors are not completely defined. Further, the measurement of inflammaging also remains vague in clinical settings. Till now, limiting inflammaging by calorie restriction, physical activity along with small-molecule inhibitors is the most reported approach [16,17,18,19]. Although implementation of a healthy lifestyle might support in slowing down and delaying the aging process, scientists are endeavoring to understand the possible regulatory mechanisms and discover potential anti-inflammaging molecules by examining several functional foods, dietary nutraceuticals, and pharmacological compounds. In particular, nutritional interventions from natural and synthetic sources in delaying the inflammaging and effective strategies in mitigating the effects of inflammaging and its progress are gaining interest and acquiring new insights [20,21,22]. In this review, the key inflammatory changes that occur during inflammaging, the available literature, clinical studies, and patents on inhibitory molecules in mitigating or delaying inflammaging from natural and synthetic nutrient compounds, were discussed.

## 2. Mechanisms of Inflammaging

Earlier studies highlighted various mechanisms that involve the activation of innate immunity together with a rise of proinflammatory mediators and the chronic inflammatory process with aging and oxidation-inflammation theory of aging [23,24,25,26]. The primary feature based on these theories highlights an increase in the body’s pro-inflammatory status with advancing age. In the following sections, the key inflammaging theories are briefly summarized to elucidate the inflammaging mechanisms with respect to the use of nutrients as anti-inflammaging therapeutic targets.

### 2.1. Cytokines in Inflammaging

Mounting evidence suggests that inflammaging is associated with elevated levels of pro-inflammatory cytokines such as TNF-α, IL-6, IL-1, interferon-γ (IFN-γ), and IL-18 [8,9,10]. An increased amount of TNF-α, IL-6, and CRP has been found in elderly patient’s serum and strongly correlates with mortality, morbidity, and frailty [27,28]. Studies also indicated that an increase in IL-1, IL-6, TNF-α, and PGE2 in circulation leads to the proinflammatory status in elderly patients and IL-6 can be referred to as one of the important predictive markers for inflammaging [14,29,30]. Notably, this proinflammatory status lead by cytokines creates an inflammatory environment in the tissues and organs, which plays a crucial role in inflammaging [31].

Maintaining a balance between pro- and anti-inflammatory status has been long linked with aging and longevity. Genetic polymorphism in inflammatory cytokines gene might play a significant role to balance and maintain this inflammatory status. A study among an Italian cohort showed an increase in the frequency of -174C single nucleotide polymorphism (SNP) in the IL-6 promoter region in male centenarians while there was an increase in the frequency of -1082G SNP at the 5′ flanking region of the IL-10 gene coding sequence. On the other hand, an increase in the +874A SNP at the IFN-γ gene was found in the female centenarians [32]. Further, genome-wide association study (GWAS) study among Han Chinese centenarians further confirms SNP mapping in IL-6 gene locus (rs2069837) was associated with longevity [33]. Further, a previous study also revealed that polymorphism in the C/G 174 on the IL-6 coding gene is associated with alterations with IL-6 serum concentration and corresponding IL-10 level [34].

### 2.2. Oxidative Stress in Inflammaging

The free radical concept of aging, wherein accumulation of excessive reactive oxygen species (ROS) produced in our cells is one of the major causative factors of oxidative stress [35]. Moreover, inflammation leads to increased levels of ROS, inducing consistent chronic oxidative stress. Increased ROS formation with weakened oxidative defense has been linked with inflammaging as both ROS and inflammaging exhibit mutual stimulatory roles [36]. Further, oxidative phosphorylation is accelerated among the elderly, which results in the accumulation of oxygen metabolites. An increasing amount of oxygen metabolites can damage the cellular components such as DNA, RNA, lipids, and proteins, thus affecting the cellular homeostasis [37]. Additionally, accumulating oxygen metabolites increases cell membrane porosity and reduces adenosine triphosphate (ATP) levels, which can accelerate the cellular aging process [38]. The functional capacity of the immune cells, individual lifespan, and the redox state are correlated and provide the oxidation-inflammatory theory of aging [24]. This theory suggests that a significant reduction in oxidative stress with the administration of potential antioxidant nutrients possessing anti-inflammatory effects in regular diet might delay the aging process and increase longevity [39].

### 2.3. Cellular Senescence in Inflammaging

Cellular senescence is best characterized by reduced cell proliferation and cell cycle arrest [40]. Cellular senescence can be caused by shortening of telomere, DNA damage, mitochondrial DNA damage, danger-associated molecular pattern (DAMPs) exposure, epigenetic modifications, point mutation, and also by stress-related signaling pathways. Senescent cells are accumulated in different organs and tissues, which increases exponentially with aging [41]. Even though no prominent marker for senescence exists, a cyclin-dependent kinase inhibitor 2A (P16INK4A) encoding gene CDKN2A was found to possess the strongest association and, therefore, was used as a common biomarker to characterize the cellular senescence [42]. A GWAS study showed that aging-associated diseases such as cardiovascular diseases (CVDs) and T2D are associated with SNPs located near senescence and inflammation [43,44]. Additionally, a common variant rs2811712, which is close to CDKN2A, was found to be associated with poor physical function in elderly [45]. Interestingly, a study suggests that the elimination of p16^Ink4a^-positive senescent cells decreases aging-associated diseases and increases lifespan [46,47]. DNA damage caused by telomere shortening results in replicative senescence and aging-associated diseases [40,48]. DNA damage response (DDR) increases pro-inflammatory status by activating adjacent cell DDR. The overall DDR in immune cells might accelerate the inflammaging process [49].

### 2.4. Autophagy in Inflammaging

Autophagy is a common physiological process of recycling and clearing detrimental substances such as misfolded proteins and damaged organelles to maintain cellular homeostasis [39]. However, the imbalance between production and cellular clearance of cell debris and misfolded protein disrupts cellular homeostasis, which may lead to inflammaging [40]. With the progression of age, cellular autophagy is believed to decline [41]. As a result, there is an increase in the accumulation of detrimental substances, production of ROS, and proinflammatory responses, which accelerates the process of aging [42]. Although several pathways are involved in the imbalance of clearing cellular debris, increased production of ROS can result in activating NF-κB signaling and consequent inflammatory responses [50]. A study has linked NLRP3 inflammasome with inflammaging by functional decline [43]. Excessive accumulation of DAMPs can result in the activation of NLRP3 inflammasome, which eventually causes the production of cytokines like IL-1 and IL-18. A study suggests excessive amount of IL-1 and IL-18 are responsible for several aging-associated diseases such as CVD and T2D [16].

## 3. Anti-Inflammaging Nutrient Compounds from Natural and Synthetic Sources

Increasing evidence suggests that natural and synthetic nutrient interventions have major influence in delaying inflammaging and aid in the prevention of inflammation-associated ARDs. In the following section, we reviewed the selected nutrient compounds that possibly target inflammaging by regulating various inflammation and aging intertwined pathways. A list of selected nutrient compounds reviewed indicating the sources, experimental models, and mechanisms, was shown in Table 1.

### 3.1. Resveratrol

**Figure 1:**
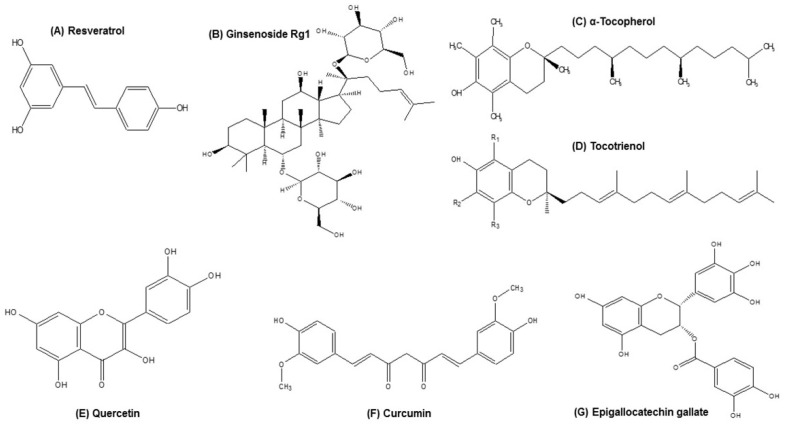
Chemical structures of selected nutrient compounds targeting inflammaging.

Resveratrol (trans-3,4,5-trihydroxystilbene, Figure 1A) is a naturally occurring polyphenol abundantly present in many sources such as red variety of grapes, peanuts, blueberries, pines, and rhubarb. In traditional Chinese and Japanese medicine, resveratrol has been used for a long time in the form of extract from *Polygonum cuspidatum* [51]. Pharmacologically, resveratrol has shown strong antioxidant, anti-inflammatory, neuroprotective, anti-microbial, and anti-cancer effects in a number of studies [52,53,54,55].

Moreover, a strong line of evidence suggests that supplementation with resveratrol can delay the aging process [56]. Liang and group reported that resveratrol can inhibit ROS in vitro in rabbit articular chondrocytes. The authors indicated that pretreatment with resveratrol (100 µM) significantly lowered the sodium nitroprusside-induced ROS. The study also revealed that resveratrol can contribute to a reduction in apoptosis due to its ROS scavenging effects [57].

Previously, resveratrol was reported to work through the activation of silencing information regulator 1 (SIRT1), which can inhibit NF-κB-regulated inflammatory cytokines [58]. Further, another study also claimed that resveratrol mediated an increase in the expression of PPAR-γ, and SIRT1 leads to downregulation of inflammation [59]. Another study suggests that activation of SIRT1 by resveratrol can also downregulate TNF-α-induced IL-1β and IL-6 expression in fibroblast cell line 3T3, and reduces phosphorylation of rapamycin (mTOR) and S6 ribosomal protein (S6RP) [60]. Overall, these data suggest that resveratrol possesses a significant effect against inflammation through SIRT1 pathway, which is also a key signaling pathway in regulating lifespan and thereby the inflammaging process [61].

Interestingly, resveratrol is able to induce a telomerase maintenance factor WRN helicase without affecting cell proliferation, and thus, might contribute to prevent telomerase dysfunction [62]. Further, resveratrol dose-dependently diminished cellular senescence, proving its capability in delaying cellular senescence in endothelial progenitor cells, thus increasing the telomerase activity [63]. In a study by Tung et al., the effect of resveratrol on aged mice was investigated [64]. In their study, young (2 months), adult (12 months), and old (18 months) mice were fed with resveratrol (24 mg/kg/day) and later the progression of proinflammatory markers were investigated in the liver tissues. Data revealed that the proinflammatory cytokines such as IL1β, IL-6, IL-17, and TNF-α increased with age in mice liver. Pretreatment with resveratrol attenuated the increased IL-1β and TNF-α protein and mRNA expression in only old mice. In addition, the authors observed an increase in the amount of ASC (NLRP3 inflammasome component apoptosis-associated speck-like protein containing a CARD), caspase-1, and NALP-3 (NACHT, LRR and PYD domains-containing protein 3) in aging mice, which was also reversed by resveratrol. This study further proves the possible effect of resveratrol in inhibiting inflammaging.

Another study conducted on aged female mice by Jeong et al., further strengthened the links between resveratrol and inflammaging, where pretreatment of 0.1 mg/kg resveratrol in aged female mice reduced IL-1β and TNF-α levels. In addition, treatment of resveratrol also inhibited stroke-induced brain injury and inflammation [65]. Taken together, these studies suggest that resveratrol has multiple targets in attenuating aging-associated chronic inflammatory pathways. Thus, resveratrol can be a useful nutrient compound against inflammaging and inflammaging-associated disorders.

### 3.2. Ginseng (Panax Ginseng)

Korean red ginseng, scientifically known as Panax ginseng (P. ginseng), has been traditionally used to treat several ailments including immune-related disorders such as inflammatory and autoimmune diseases. It is regarded as a miracle herb found mostly in Korea and China with several active component types of ginsenosides and acidic polysaccharides [66,67]. Recently, the polysaccharides (30 mg/kg) from P. ginseng berries were reported to exert protection against immunosenescent effect in aged mice by reducing the IL-6 and IL-2 cytokine expression. The authors indicated that ginseng-derived polysaccharides might be useful as antiaging agents by regulating the inflammatory immune functions [68]. In another study by Wang and group, the ginsenoside Rg1 from P. ginseng (Figure 1B) attenuated the excessive production of inflammatory cytokines in D-galactase (D-gal)-induced aging mouse. The authors investigated the testicular senescence changes in D-gal-induced aging mice by evaluating the anti-inflammatory parameters including tumor necrosis factor-α, interleukin (IL)-1β, and IL-6 in testicular tissues. Ginsenoside Rg1 (20 mg/kg/day) treated for 2 weeks after D-gal administration reduced the levels of inflammatory cytokines significantly, indicating the anti-inflammaging role of Rg1 on male reproductive function [69].

### 3.3. Tocotrienol

Vitamin E, a common nutrient found in seeds, cooking oils, nuts, and most foods, is a liposoluble antioxidant that contains two groups, α-tocopherol and tocotrienol (Figure 1C,D). Tocopherol has been studied extensively and is well known for its antioxidant protective role. Tocotrienol is a newer discovery compared to tocopherol and is mostly found in many edible plants such as palm tree, annatto, and achiote tree [70,71]. Several studies with tocotrienol suggest its superiority in the antioxidant activity compared to tocopherol [72]. Although tocopherol and tocotrienol exhibit the same chromanol head, they are structurally different in the hydrophobic tridecyl chain saturation. Tocopherol contains saturated phytol chains whereas tocotrienol contains unsaturated farnesyl isoprenoid chains. Both tocopherol and tocotrienol have four homologs each (alpha, beta, gamma, and delta) and depend on the location and number of methyl groups on the chromanol ring [71].

In a study by Ahn et al., the authors indicated that gamma tocotrienol inhibited IκB-α kinase activation, thus leading to the subsequent inhibition of NF-κB activation [73]. Moreover, Wong et al., investigated the anti-inflammatory role of different homologs of tocotrienols and alpha-tocopherol. The in vivo study suggested that delta and gamma tocotrienol comprises a significant improvement in inflammation, heart, and liver function in rats [74]. Inflammaging has been broadly correlated with IL-6 and CRP. Interestingly in a study by Yam and group, the authors identified that tocotrienol can significantly inhibit IL-6 production in lipopolysaccharide (LPS)-stimulated RAW264.7 macrophages [75]. Also, this study reported the reduction of cyclooxygenase (COX)-2, prostaglandin (PG)-E2 by tocotrienol. In the same line of investigation, a report by Qureshi and colleagues showed inhibition of TNF-α by tocotrienol in Raw264.7 cells. They also demonstrated that in BALB/c mice serum, TNF-α levels were significantly decreased by the treatment of tocotrienol with doses of 1–10 µg/kg [76]. In a study on T2D individuals supplemented with alpha tocopherol for 3 months, a significant reduction in their CRP and IL-6 plasma levels was shown [77]. CRP and IL-6 are two of the most common cytokines found in individuals with inflammaging. Since tocotrienol targets different pathways involved in inflammaging, it might serve as a potential anti-inflammaging candidate.

### 3.4. Quercetin

Quercetin (2-(3,4-Dihydroxyphenyl)-5,7-dihydroxy-4H-1-benzopyran-4-one, Figure 1E) is a well-studied flavonol containing several health beneficiary properties [78]. Quercetin is abundantly and widely found in various dietary sources such as apples, grapes, tomatoes, onions, berries, and brassica vegetables. Quercetin is also available in natural herbs including *Hypericum perforatum*, *Ginkgo biloba*, and *Sambucus canadensis* [79,80]. Quercetin exhibits a wide range of properties that can play a potential role in immunity and shows overall health benefits through anti-inflammatory, anti-oxidant, anti-viral, and anti-carcinogenic activities [81]. In murine macrophages, a significant reduction of TNF-α production after inducing LPS and in glial cells a reduction in mRNA of TNF-α has been reported [82,83]. Also, in a study conducted in lung A549 cells, quercetin showed a reduction in IL-8 [84]. This flavonol also suppressed the IL-1-stimulated selective release of IL-6 in mast cells significantly [85].

Independent in vitro studies with RPE and THP1 cell line demonstrated inhibition of oxidative stress by quercetin [86,87]. In addition, a recent study conducted by Bao et al. demonstrated that hydrogen peroxide (H_2_O_2_)-induced oxidative stress is significantly inhibited by the treatment of quercetin in rat pheochromocytoma (PC12) cells. This study also showed a protective effect of quercetin in H_2_O_2_-induced cell death as quercetin significantly reduced lactate dehydrogenase (LDH) from PC12 cells [88]. Interestingly, recent studies shined a light on the effect of quercetin on aging. A study by Saul et al. demonstrated that quercetin can improve lifespan by 15% in *Caenorhabditis elegans* [89]. As oxidative stress is a leading factor for developing inflammaging and quercetin is known to be widely used as an antioxidant due to its potent ROS and reactive nitrogen species (RNS) scavenging effects, quercetin can be considered as a valuable nutrient in controlling oxidative stress-mediated inflammaging.

### 3.5. Curcumin

Curcumin (Figure 1F) is the major component of turmeric and is a very commonly used spice and coloring agent in various foods throughout the world. It is a yellow pigment that is naturally found in the rhizome of *Curcuma longa* from the herb family *of Zingiberaceae* [90]. Curcumin has long been used as a traditional medicine in India and other Asian countries for many diseases. Curcumin exhibits anti-oxidant, anti-inflammatory, and anti-carcinogenic properties, which make it an attractive source as a potential nutrient supplement. The anti-inflammatory function of curcumin has been well established in the last few decades. Curcumin can potentially reduce the activity of several inflammation-related transcription factors including NF-κB, AP-1, signal transducer and activator of transcription (STAT), and hypoxia inducible factor-1 (HIF-1). Curcumin inhibits NF-κB activity by inhibiting P65 translocation to the nucleus and suppressing IκB-α degradation. It has also been demonstrated by several authors that the inhibition of NF-κB transcription factor by curcumin leads to reduction in TNF-α, IL-6, and COX-2 [90].

A recent study showed that the anti-inflammatory activity of curcumin through NF-κB depends on its oxidized form. The authors showed inactivation of curcumin electrophiles through the pretreatment of N-acetyl cysteine, which is a precursor of glutathione (GSH). Oxidative stress is involved in several ARDs and the protective effect of curcumin against oxidative stress can favor its use as an anti-inflammaging agent [90]. Additionally, several in vivo studies conducted on mice and rats showed a significant inhibition in oxidative stress. For instance, Sood et al., showed that aluminum-induced oxidative stress has been reduced by free curcumin, as curcumin prevented depletion of GSH [91]. Another study showed that curcumin prevents the increase in malondialdehyde (MDA) and nitrates in the hippocampus of LPS-induced neurobehavioral and neurochemical deficits Swiss albino mice [92].

Curcumin also exhibited its potency against neuroinflammation-mediated aging in in vitro and in vivo models of diseases such as Alzheimer’s disease (AD) and Parkinson’s disease (PD) [93,94]. Further, Rastogi et al., showed that curcuminoid is capable of inhibiting age-associated mitochondrial impairment in rats [95]. The authors indicated that 100 mg/kg orally-treated curcuminoid inhibited age-associated enzymes such as NADH dehydrogenase, cytochrome c oxidase, Complex I, and total ATP content. The curcuminoid also suppressed neuronal nitric oxide synthase (NOS) in mitochondria. Furthermore, a study on mice showed the ability of curcumin on age-related cognitive dysfunction, where curcumin significantly inhibited oxidative stress [96]. The pleiotropic activity exhibited by curcumin by regulating various inflammatory mechanisms in aging models might open the door for curcumin to be developed as a potential anti-inflammaging agent.

### 3.6. Epigallocatechin-3-Gallate

Epigallocatechin-3-gallate (EGCG, Figure 1G) is the most abundant catechin found in green tea with immense medicinal benefits [97,98]. Even though green tea contains other catechins, EGCG comprises 50–60% among them all. As a catechin, EGCG contains dihydroxyl or trihydroxyl substitutions on the B ring and the m-5,7-dihydroxyl substitutions on the A ring. Few studies suggest that the B ring is the principal site for the antioxidant property of EGCG, and the polyphenolic structure is believed to be responsible for the ROS quenching [99,100]. EGCG contains several health beneficiaries such as anti-oxidant, anti-inflammatory, and anti-cancer effects. EGCG comprises anti-inflammatory effects through the inhibition of gene expression of TNF-α, COX-2, and iNOS [101]. Studies also showed that EGCG can directly suppress NF-κB and AP-1 in human ECV304 cells [102].

In a study on RAW264.7 cells, EGCG showed its inhibition through TLR4-mediated MyD88 and TRIF signaling pathways [103]. EGCG also exerts antioxidant activity by reducing ROS and suppressing oxidative stress by activating nuclear factor erythroid-2 like factor-2 (Nrf-2) transcription factor [104]. An in vivo study by Nui et al. suggested that 25 mg/kg of EGCG extends lifespan in rats [105]. In a recent study by the same group, 90 male Wistar rats were challenged with a high fat diet and 60 mg/kg EGCG throughout their lifetime. EGCG extended lifespan significantly among high fat diet-fed male rats. Further, EGCG significantly decreased the IL-6, TNF-α level in serum of rats and suppressed the NF-κB expression. Additionally, the authors also determined the level of ROS in the serum of EGCG-induced rats. The ability of EGCG to suppress inflammation and ROS in experimental models might contribute to increased lifespan [106]. In addition, a cohort study in China supported the notion that consumption of green tea increases lifespan [107]. Based on the overall studies, EGCG might be developed as a promising anti-inflammaging nutrient.

### 3.7. Huperzine A

**Figure 2:**
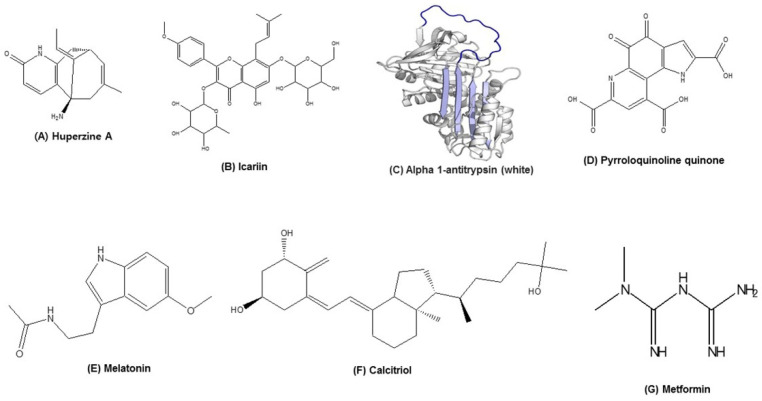
Chemical structures of selected natural and synthetic nutrient compounds targeting inflammaging.

Huperzine A (HupA; Figure 2A) is a naturally occurring Lycopodium alkaloid isolated from the Chinese popular traditional medicine *Huperzia serrata*. HupA is a potent, reversible, and selective inhibitor of acetylcholinesterase enzyme, and has been used in China since long as a medicament for various disorders such as strains, swellings, contusions, schizophrenia, and fever [108,109]. Further, HupA is believed to have significant effects against cognitive deficits and is an approved drug for treating AD in China [110]. Besides that, several studies have been done to investigate the effect of HupA on multiple directions/pathways. Wang et al., reported that the effect of 0.1 mg/kg HupA intraperitoneal injection decreased neurological deficits in a rat model of transient focal cerebral ischemia model. More importantly, the authors also found that HupA inhibited nuclear translocation of NF-κB and overexpression of several proinflammatory factors [111].

A study conducted by Ruan et al. showed that HupA exhibited hepatoprotective activity based on its anti-inflammaging effects in D-galactose (D-gal)-treated rats [112]. D-gal (300 mg/kg s.c.) administered rats showed increased oxidative damage, hepatic senescence, nuclear factor-kappa B (NF-κB) activation, and inflammatory response. Co-administration with HupA A (0.1 mg/kg s.c.) for 8 weeks significantly attenuated the D-gal-induced changes and also exhibited anti-inflammaging effects by hepatic replicative senescence inhibition in experimental rats. Additionally, the study also demonstrated that the suppression of TNF-α, IL-6, and IL-1β expression level by HupA is via the inhibition of NF-κB pathway. Overall, the results provided a scientific basis on the role of HupA as a potential anti-inflammaging nutrient.

### 3.8. Icariin

Icariin (ICA; Figure 2B) is a flavonoid and bioactive component of *Herba epimedii,* which is a popular Chinese medicine for treating various diseases [113]. *H. epimedii* is traditionally used for several age-associated diseases such as CVDs, osteoporosis, neurological disorder, and sexual dysfunctions [114]. ICA, the main component of total flavone of *Epimedium* (TFE), was extensively studied and is well known as an aging intervention drug. In vitro studies on human diploid fibroblasts and *C. elegans* showed that ICA extends the lifespan by protecting the length of telomere [115,116]. An in vivo study also confirmed that administration of ICA from 12 months of age increased the health span and mean lifespan in mice. The study showed that the ability of ICA in decreasing oxidative stress and DNA damage might contribute to extended health and life span in mice [117]. Further, ICA was well reported to downregulate the NF-κB pathway, thus contributing to lowering inflammatory pathways. Recently, Chen et al. showed that the downregulation of NF-κB pathway by ICA might be responsible for the upregulation of SIRT6. The study confirmed the inhibition of TNF-α and IL-6 expression by the treatment of ICA (0.02% ICA for 3 months in feed). Additionally, the authors predicted that the effect of ICA in regulating NF-κB pathway through SIRT6 might inhibit cardiac inflammaging, indicating a novel link between the SIRT6 (a regulator of aging) and NF-κB (a regulator of inflammation) and the synergistic effect of ICA in preventing inflammaging [118].

### 3.9. Blueberry

Blueberry fruit from Ericaceae family contains enormous functional phytoconstituents including polyphenolic and flavonoid components. Pharmacological studies revealed that blueberry possesses anti-diabetic, anti-inflammatory, anti-tumor, and neuroprotective effects [119]. Evidence suggests that blueberry exhibited strong protection against inflammation in pre-clinical and clinical evaluations [120,121,122]. In a study by Goyarzu et al., the flavonoid-rich diets including the anti-oxidant rich blue berry were well reported to prevent cognitive impairment associated with inflammaging in animal studies [123]. Aged Fischer-344 rats were supplemented with 2% blueberry diet for 4 months, and the cognitive parameters and biochemical parameters were evacuated in comparison with aged control groups. The authors found better performance in cognitive tasks in comparison with aged control. The inflammatory signaling NF-κB expression levels in four brain regions in blueberry-supplemented aged rats showed lower levels when compared with aged control groups. The impaired object recognition memory scores were correlated with high levels of NF-κB in aged control groups, indicating that blueberry-supplemented rat diet may retard brain inflammaging in aged rats.

### 3.10. Prune (Prunus spinosa L.)

*P. spinosa,* commonly known as blackthorn from Rosaceae family, is majorly found in Asia, Europe, and the Mediterranean. *P. spinosa* has been used traditionally for centuries as a diuretic, laxative, and antispasmodic, and to prevent inflammatory conditions [124,125]. In a recent report, the wound healing effects of *P. spinosa* fruit extract were evaluated against LPS challenge in young and aged (senescent) human umbilical vein endothelial cells (HUVECs) based on its anti-inflammatory effects. The authors found that *P. spinosa* exerted (40 µg/mL) potent anti-oxidant and anti-inflammaging ability in older cells when compared with younger cells by downregulation of inflammatory markers including IL-1 receptor associated kinase 1 (IRAK-1) and IL-6. Further, the *Prunus* extract (400 µg/mL) increased the life and health span in *C. elegans*. Overall, the study indicated that *P. spinosa* fruit extract might enhance wound healing capacity in senescent conditions, thereby improving the quality of life in aging populations [126].

### 3.11. Alpha-1 Antitrypsin (AAT)

Alpha-1 antitrypsin (AAT, Figure 2C) is an acute phase glycoprotein produced mainly in liver that protects lungs [127,128]. AAT works as a protease inhibitor on trypsin, chymotrypsin, thrombin, and elastase. AAT is one of the most abundant serine protease inhibitors found in circulating human blood and is able to inhibit proteinase 3 and cathepsin G. However, if human alpha-1 antitrypsin (hAAT) is modified by NO, it can work as a cysteine protease inhibitor [129]. Even though hAAT is abundant in circulation, its expression can be increased by IL-6 and LPS treatment [130]. Further studies by Petrache et al. showed that AAT can inhibit apoptosis in alveolar cell. This study also confirmed that hAAT is able to decrease caspase-3 and oxidative stress at a cellular level [131]. Many studies regarding AAT further proved the role of hAAT in ARDs such as stroke, T1D, and rheumatoid arthritis. A study by Wang et al. showed that AAT is responsible for the suppression of instant blood-mediated inflammatory reaction (IBMR). The authors indicated that significantly lower TNF-α levels, lymphocytic infiltration, and decreased nuclear factor activation were observed in AAT-treated mice compared to control mice. They suggested the potent anti-inflammatory role of AAT is due to the inhibition of JNK phosphorylation [132]. AAT exhibits an anti-inflammatory, immune-regulatory, and cytoprotective role against many diseases. A recent study by Yuan et al. provided direct evidence of hAAT possessing therapeutic potential against inflammaging [133]. In their study, they found that hAAT overexpressing *Drosophila* cell line demonstrated longer lifespan than the control cell line. Interestingly, the identified two aging-associated genes *Relish* and *Diptericin* were significantly lower in the hAAT overexpressing cells. Moreover, RNA sequence analysis showed a significant decrease in the NF-κB-regulated innate immunity genes in hAAT overexpressing Drosophila cell lines.

In another line of evidence, RNA seq analysis also showed a decrease in inflammation-related genes. Experiments with human cell line confirm that hAAT treatment was able to suppresses senescence-associated secretory phenotype (SASP), as it inhibited the IL-6 and IL-8 in X-ray-induced senescence cells. Further, hAAT is an FDA-approved drug with a proven safety profile, making it a potential candidate in inflammaging and aging-associated disorders.

### 3.12. Pyrroloquinoline Quinone 

Pyrroloquinoline Quinone (PPQ; Figure 2D), a quinone nutrient substance widely available in the plant foods and animal tissues, helps in scavenging harmful free radicals, reduces oxidative stress, and suppresses the inflammation markers such as serum CRP and IL-6 [134,135]. A study by Zhang et al. was undertaken to assess the anti-aging potential of PQQ in HepG2 cell cultures. The authors concluded that PQQ (10–30 μM) contributes to damage repair and delays cell senescence [136]. In another study, PQQ delayed inflammaging induced by TNF-α, and abated inflammatory cells as well as inflammatory cytokine infiltration. PQQ also partly delayed the premature ageing phenotype; promoted cell proliferation; decreased the expression of cell cycle arrest protein p16, P19, P21, P27, and P53 expression; and promoted the expression of longevity genes *SIRT1* and *SIRT3* [15,118,136].

In a recent study, PPQ ameliorated D-gal-induced oxidative stress and inflammatory response, resulting in cognitive improvements in mouse. PPQ inhibited the hippocampal MDA and increased the SOD expression, thereby exerting strong antioxidant effects in D-gal-induced mice. Further, PQQ attenuated the increased inflammatory factors (IL-2 and IFN-γ) and the production of prostaglandin E2 (PGE2) in D-gal-induced mice [137]. More recently, the effect of PQQ in cultivated human embryonic lung fibroblasts WI-38 cells with or without TNF-α was studied to establish an inflammaging model in vitro. Data revealed that compared with TNF-α stimulation alone, PQQ (150 nmol/L) showed less SA-β-gal-positive cells, indicating that PQQ attenuated TNF-α-induced inflammaging damage. The authors concluded that PQQ delayed TNF-α-induced cellular senescence and had anti-inflammaging properties [138]. Based on these findings, it is clear that PQQ may influence the generation of pro-inflammatory mediators, including cytokines and prostaglandins, during the aging process, and provides evidence that PPQ might be beneficial in preventing cognitive deficits during the inflammaging process.

### 3.13. Melatonin

Melatonin (Figure 2E), a naturally occurring hormone from the pineal gland in the brain, has also been widely identified and qualified in various foods from fungi to animals and plants. Melatonin concentration in human serum could significantly increase after the consumption of melatonin-containing food. Melatonin exhibits many bioactivities, such as antioxidant activity anti-inflammatory characteristics, boosts immunity, anticancer activity, cardiovascular protection, anti-diabetic, anti-obese, neuroprotective, and anti-aging activity. Melatonin levels decrease in the course of senescence and are more strongly reduced in ARDs such as coronary heart disease and T2D [139]. The role of melatonin, a highly pleiotropic regulator molecule in the antiaging mechanism associated with inflammaging, was well studied in aged organisms and senescence-accelerated animals [140]. Melatonin behaves under conditions of low-grade inflammation, especially in inflammaging. Melatonin exerts a broad spectrum of effects on physiological functions of relevance to aging, such as metabolic sensing; mitochondrial modulation and presumably also proliferation; antioxidative protection of biomolecules and subcellular structures, in particular, mitochondria; and immunological actions implicated in both the combat against foreign antigens and inflammaging. In different senescence-accelerated prone studies on various organ tissues in male mice and in aged ovariectomized female rat liver, melatonin in the dose range of 1–10 mg/kg/day for 1–3 months downregulated proinflammatory cytokines such as TNF-α, IL-1β, and IL-6, and upregulated the anti-inflammatory IL-10, indicating its role in attenuating inflammaging and ARDs [141,142,143,144].

### 3.14. Calcitriol

Calcitriol (1,25-dihydroxycholecalciferol, Figure 2F) is the inactive form of vitamin D, which is activated by activating enzyme 1 alpha-hydroxylase and converting into 1,25-dihydroxyvitamin D3 (1,25VD3) or vitamin D hormone calcitriol [145]. It is also known as the main circulatory form of vitamin D, which is used as a marker for the evaluation of physiological vitamin D levels. Vitamin D is known for its activity against inflammation and aging-associated diseases. A study on Raw264.7 macrophage cells showed 1,25VD3 is able to downregulate pro-inflammatory gene expression such as COX-2, NF-κB, and AKT [146]. Additionally, a different study showed that CD40 ligand-induced pro-inflammatory cytokines IL-1β and TNF-α are suppressed by the co-treatment with 1,25VD3 [147].

In a double-blinded, placebo-controlled, randomized clinical trial, patients with cystic fibrosis supplemented with cholecalciferol demonstrated that vitamin D suppressed two inflammatory cytokines, IL-6 and TNF-α [148]. Moreover, two different randomized control trials among diabetes patients who were given vitamin D as supplementation and calcium demonstrated a significant decrease in IL-6, IL-1β, TNF-α, and CRP in the serum level of the patients [149,150].

Further, in an in vivo study conducted by Wang et al., a model of *Porphyromonas gingivalis*-infected *db/db* mice was used. The group demonstrated that inflammaging occurred in these diabetic mice, which was measured by increased SASP, increased senescent cells, and periodontal destruction. Interestingly, the induction of 1,25VD3 significantly decreased the serum SASP and periodontal condition. The expression of NF-κB, IL-1β, and STAT-3 was also suppressed significantly in gingival tissue. Additionally, the suppressors of cytokine signaling 3 (SOCS3) level were also decreased with the administration of 1,25VD3 [151]. SOCS3 suppresses Janus kinase (JAK) and signal transducer and activator of transcription (STAT) pathway-mediated inflammatory signaling. Surprisingly, aging increased SOCS-3 expression in the hypothalamus. Thus, 1,25VD3-induced attenuation of SOCS3 might be responsible for the decrease in inflammaging through the NF-κB pathway [152,153].

### 3.15. BaZiBuShen (BZBS)

In a recent study, a Chinese over-the-counter herbal medicament named BZBS containing various natural herb extracts was investigated for sperm quality and fertility in an aging male mouse model [154]. The prescription contained Semen Cuscutae, Fructus Lycii, Epimedii Folium, Fructus Schisandrae Sphenantherae, Fructus Cnidii, Fructus Rosae Laevigatae, Semen Allii Tuberosi., Radix Morindae Officinalis, Herba Cistanches, Fructus Rubi, Radix Rehmanniae Recens, Radix Cyathulae, Radix Ginseng, Cervi Cornu Pantotrichum, Hippocampus, and Fuctus Toosendan, which are approved by China FDA (No. B20020585). In their study, aged mice were induced by D-galactose (D-gal) and NaNO_2_ for 65 days and the inflammatory signaling pathways were analyzed in testes tissues. Data revealed that treatment with BZBS (0.7, 1.4, and 2.8 g/kg/day) to D-gal- and NaNO_2_-induced mice alleviated the increased levels of TNF-α secretion, NF-κB activation, and iNOS expression in aging mice testes tissues. Further, BZBS also modulated the Sirt6 expression in the testes of aging mice. The authors concluded that BZBS rescued the altered testicular morphology and sperm quality in rapidly aging mice induced by D-gal- and NaNO_2,_ possibly via regulation of inflammatory Sirt6/NF-κB signaling, indicating that the Chinese prescription BZBS can be a promising candidate in ameliorating aging-associated inflammatory testicular damage [154].

### 3.16. Egg Yolk

Mounting evidence suggests that eggs are rich in biologically effective constituents and shows an immense role in the prevention of chronic infectious diseases. Eggs contain micro and macronutrients including zinc, selenium, retinol, and tocopherols, and low amounts of carbohydrates. Earlier reports revealed that eggs, particularly non-fertilized egg yolk, possess immunomodulatory, anti-inflammatory, and analgesic effects [155]. In a study by Cunill et al., patented egg yolk (PEY) was compared with commercial egg yolk for it anti-inflammaging properties. Mouse RAW 264.7 macrophage cells lines in vitro and LPS (2.5 mg/kg dose, i.p.) administered Wistar rats in vivo were treated with PEY and commercial egg yolk (2000 mg/kg) to two different groups, and the serum levels of cytokines including IL-1β, TNF-α, and MCP-1, were evaluated in both groups. The authors indicated that PEY group showed a significant reduction in concentrations of IL-1 β, TNF-α, and MCP-1 when compared with the commercial egg yolk group, and concluded that the complex biomolecules present in PEY might effectively control the chronic inflammation and aging and help reduce the inflammaging progression [156].

**Table 1:** Selected nutrients from natural and synthetic sources targeting inflammaging in experimental models.

Nutrient Compounds	Source	Model (In Vitro and In Vivo)	Dose	Mechanism/s	Refs
Resveratrol	Grapes, peanuts	Aged male mice	24 mg/kg/day	↓ IL-1β, TNF-α, COX-2; ↓ ASC, caspase-1 and NALP-3	[64]
		Aged female mice	0.1 mg/kg/10 days	↓ IL-1β and TNF-α ROS scavenger	[65]
APGP	*Panax ginseng* berries	Immunosenescnece old male C57BL/6J mice	5 and 30 mg/kg/daily for 20 days	↓ IL-2, =IL-6	[68]
Ginseng Rg1	*Panax ginseng*	D-gal-induced aging mice	20 mg/kg/28 days	↓ TNF-α, IL-1β, and IL-6.	[69]
Tocotrienol	Palm tree (Palm Vit E)	LPS-induced RAW264.7 cells	10 µg/kg	↓ IL-6, NO, COX-2	[75]
	Rice bran	LPS-stimulated RAW264.7 cells	4, 8, 16 μM	↓ TNF-α	[76]
		LPS-stimulated female mice	2.5, 5.0, and 10.0 μg/kg	↓ TNF-α IL-1β, IL-6, and iNOS	[76]
Tocopherol	*all-rac*-α-tocopherol	T2D patients	1200 IU/day/3 months	↓ CRP and IL-6	[77]
Quercetin	Herbs and fruits	daf-16(mgDf50) mutant strain nematode *Caenorhabditis elegans*	200 μM	↑ lifespan	[89]
Curcumin	Turmeric	Aged Wistar rats	100 mg/kg/3 months	↓ IL-6, TNF-α, mitochondrial impairment and nNOS,.	[95]
		SAMP8 mice	20 and 50 mg/kg per day/25 days	↓ MDA; ↑ p-CaMKII and p-NMDAR1	[96]
EGCG	Green tea	Life time high fat diet fed rats	60 mg/kg/life time	↓ ROS, IL-6, and TNF-α	[106]
HupA	*Huperzia serrata*	D-gal treated rats	0.1 mg/kg/ 8 weeks	↓ TNF-α, IL-6, and IL-1β, ↓ NF-κB	[112]
ICA	*Herba epimedii*	C57BL/6 aged mice	0.02% for 3 months in feed	↑ Life span; ↓ MDA	[117]
		male BALB/c mice	0.02% ICA for 3 months	↑ SIRT6; ↓ TNF-α, ICAM-1, IL-2, and IL-6 and NF-κB	[118]
Blueberry	*Vaccinium* spp.	Aged Fischer-344 rats	2% in diet for four months	↓ NF-κB and, oxidative stress	[123]
Prune	*Prunus spinosa*	LPS-induced HUVECs	40 µg/mL	↓ IRAK-1, and IL-6	[126]
		Nematode *C. elegans*	400 µg/mL	↑ life span	[126]
PPQ	Plant foods and animal tissues	D-gal-induced mice	100 μg/kg/day/for 6 weeks	↓ IL-2 and IFN-γ	[137]
		TNF-α induced human WI-38 cells	150 nmol/L	↓ TNF-α–induced cellular senescence	[138]
Melatonin	Natural hormone, Foods	Aged ovariectomized female rat	1 mg/kg/day/10 weeks	↑ IL-10; ↓ iNOS, HO-1, IL-6, TNF-α and IL-1β	[141]
		SAMP8 mice	1 mg/kg/day/one month	↓ TNF-α, IL-1β, HO (HO-1 and HO-2), iNOS, MCP1, NFκB1, NFκB2 and NKAP	[142]
		SAMP8 mice	1 and 10 mg/kg day for one month	↓ TNF-α, IL-1β, and IL-6. ↑ IL-10.	[143]
		SAMP8 mice	1 mg/kg/day/one month	↓ TNF-α, IL-1, HO-1, and NFκB; ↑ IL-10.	[144]
Calcitriol	Vitamin D	*Porphyromonas gingivalis*-infected *db/db* mice	5 μg/kg/alternative day for 10 weeks	Regulation of NF-κB, IL-1β, STAT-3	[151]
BZBS	Herbal preparation	D-gal-induced aged mice	0.7, 1.4, and 2.8 g/kg/day for 65 days	Regulation of Sirt6/NF-κB	[154]
PEY	Eggs	LPS-induced RAW 264.7 macrophage cells.	2000 mg/kg	↓ IL-1 β, TNF-α, and MCP-1	[156]

Abbreviations: IL: interleukin; LPS: lipopolysaccharide; TNF-α: tissue necrosis factor-alpha; ROS: reactive oxygen species; NF-κB: nuclear factor-kappa B; CRP: C-reactive protein; Rice NPN: Rice natural peptide network; MCP-1: monocyte chemoattractant protein-1; nNOS: neuronal nitric oxide synthase; IFN-γ: interferon-gamma; IRAK-1: IL-1 receptor associated kinase 1; APGP: acidic-polysaccharide-linked glycopeptide; EGCG: epigallocatechin-3-gallate; HupA: huperzine A; ICA: icariin; PPQ: pyrroloquinoline quinone; BZBS: BaZiBuShen; PEY: patented egg yolk; p-CaMKII: p-calcium/calmodulin-dependent kinase II; p-NMDAR1: p-N-methyl-d-aspartate receptor subunit 1; HO-1: heme oxygenase 1; SAMP8 mice: Senescence-accelerated mice; D-gal: *D*-galactose; ↑: increased; ↓: decreased; =: no change.

## 4. Clinical Trials and Human Research on Nutrient Compounds for Possible Anti-Inflammaging Effects

Data obtained from clinical trials and research involving human subjects can be ideal in providing strong insights in developing nutrient compounds aimed at controlling inflammaging and regulation of its mechanisms. In the following section we reviewed the available clinical data and studies involving humans on nutrient compounds from natural and synthetic sources targeting inflammaging. A list of selected nutrient compounds with reported clinical trials and human research used in preventing or delaying inflammaging is shown in Table 2.

### 4.1. Metformin

Metformin (Figure 2G), derived from galegine, a natural product from the plant *Galega officinalis*, has been used in herbal medicine in Europe since medieval times. Metformin, a prescribed drug for T2D, improves glycemic control and shows clear benefits in relation to glucose metabolism and diabetes-related complications. Cohort studies suggested that metformin might work as an anti-aging molecule, as it lengthens the lifespan and has been found to inhibit inflammation [157,158,159]. Inflammaging is associated with defective autophagy that increases with age. A recent cross sectional study shed light on the mechanism of metformin in diabetes related to age-associated diseases. The study involved young lean subjects (31.2 years.) and BMI-matched older subjects (62 years.) treated with metformin, and the CD4^+^ T cells from peripheral blood were collected for various assays. The study showed that metformin (1000 mg/day) for 3 months prevented the production of Th17 inflammaging profile. Metformin improved autophagy and mitochondrial function, and thus, reduced inflammaging [160]. Moreover, metformin works in multiple ways against age and ageing-associated diseases through inhibition of ROS production [161]. Studies also showed the effect of metformin against DNA damage and cellular senescence [162,163]. Metformin showed an effect against LPS-induced NF-κB pathway, thus working against inflammation itself [162]. Since metformin is an FDA-approved drug for other conditions including diabetes with well-known safety profile, human trials in assessing the efficacy of metformin for its ability to stimulate autophagy and exhibit anti-inflammaging effects is quite beneficial.

### 4.2. Zinc

Zinc is the second most prevalent trace element in the human body and is essential for several cellular and metabolic functions and also for the immune system. The adult human body contains 2–3 g of zinc, and it is relatively known as a non-toxic. Many times zinc is given as a supplement to maintain its level in the human body in order to achieve appropriate immune function [164]. The role of zinc in anti-inflammation has been long studied, which shows the ability of zinc to reduce inflammatory cytokines. More interestingly, zinc has been shown to reduce inflammaging-associated cytokines. The conventional inflammatory pathway, NF-κB, is effected by zinc, and in the human monocyte, LPS-induced TNF-α expression is significantly suppressed [165]. A randomized, double-blinded placebo trial of zinc supplementation (45 mg/day for six months) in elderly (56–83 years) showed a significant reduction in CRP, IL-6, and TNF-α levels [166]. In a different study conducted by Jung et al., subjects aged 40 years and older showed a decrease in CRP and IL-6 in the plasma of individuals after zinc supplementation [167]. Studies also suggested that plasma zinc and CRP levels are inversely correlated in elderly patients [168]. Overall, the importance of micronutrients in nutrition such as zinc, especially in geriatric patients, is justified as it plays a significant role in healthy aging and immunosenescence.

### 4.3. Gotu Kola

Gotu kola (*Centella asiatica* Linn.), is a natural herb extensively used in traditional Ayurvedic system in India and other parts of the world to treat various ailments. In a study by Maramaldi et al., the antiinflammaging capacity of this botanical herb was studied clinically in human explants and volunteers [169]. *C. asiatica* extracts (2 mg/explant) were treated on UV-irradiation-induced human explants maintained alive and obtained from a 58-year-old Caucasian woman, and the expression of the proinflammatory cytokine IL-1α was evaluated. Further, a single-blind, placebo-controlled clinical trial on healthy volunteers (age 40–70 years) also revealed that *C. asiatica* exhibited antiaging and anti-wrinkling properties by improving the skin firmness, wrinkling, elasticity, and collagen density. The authors concluded the antiaging efficacy of *C. asiatica* might be due to its anti-inflammaging effects along with free radical scavenging and antiglycation activities.

### 4.4. Soy and Whey Proteins

Dairy and soy proteins contain various amino acids and bioactive peptides with immense nutritional values. Earlier studies indicated that soy and whey protein possess anti-oxidant and anti-inflammatory effects including mitigating chronic inflammation during aging [170]. In a crossover designed randomized, acute clinical intervention study, a fat-rich mixed meal was administered with 45 g of whey protein to obese non-diabetic aged subjects (40–68 years). The authors observed an acute suppression of inflammatory markers of low-grade inflammation including monocyte chemotactic protein-1 (MCP-1) expression in the blood samples after 240 min postprandial period [171]. Further, soy protein, which is considered to be a high nutritional protein, is well reported for its potential nutritional intervention for chronic inflammatory conditions in experimental studies [172]. Soy protein was documented to attenuate chronic inflammation through regulation of the NF-κB signaling pathway and cytokine production in *mdx* mice [173]. In a randomized, double-blind, placebo-control, clinical trial involving 131 healthy older women (60 years.), the long term effects (1 yr.) of soy protein (18 g) on the serum lipids and inflammatory markers were evaluated. Data showed that after 1 year of soy protein administration, a significant reduction in IL-6 baseline when compared to control group was observed [174]. Based on the promising nutritional interventions of dietary proteins, future studies involving detailed clinical studies focusing on inflammaging and its associated diseases should be investigated.

### 4.5. Black Rice

Recently, food-derived bioactive peptide nutrients and optimized diet habits have been emerging as potential sources for the prevention and treatment of several diseases including attenuating age-related inflammation by regulating the balance between pro- and anti-inflammaging [175,176,177]. In a recent report, rice-derived functional ingredient natural peptide network (rice NPN) significantly reduced TNF-α secretion in human macrophages stimulated by lipopolysaccharide in vitro [178]. Black rice (*Oryza sativa* L. var. japonica), a staple food for Asian populations since ancient times, was well documented for it antioxidant and anti-inflammatory properties due to its rich bioactive functional peptides [179].

A randomized, double blinded, parallel group, placebo-controlled clinical trial with participants (males and females, *n* = 30) aging between 65–75 years was conducted to study the anti-inflammatory effects of rice NPN. Clinical data revealed that 4-week supply of rice NPN (10 g) was found to help improve the physical challenges measured by hand grip test, repeated chair stand test, and short physical performance battery (SPPB) test score in inflammaging populations when compared with placebo group. The inflammation-associated aging-mediated altered glucose, serum LDL, and HDL levels were restored, providing the efficacy data of rice NPN against the inflammaging process both in vitro and in clinical settings.

### 4.6. Mediterranean Diet

Evidence suggests that the Mediterranean diet (MedDiet) showed beneficial effects by positively influencing the ageing hallmarks and helps in mitigating age-related disease and increasing longevity [177]. In a study by Martucci and group, the nutritious supplements provided by (MedDiet) rich in whole-grain cereals, vegetable, fruits, legumes, fish, olive oils, and nuts aided in neurohormetic and neuroprotective effects. MedDiet also regulates the balance between pro and anti-inflammaging conditions. The authors indicated that hormetic interventions by both nutritional and physical activity, control the inflammaging processes by decreasing the senescent cells accumulation [176]. Further, data from the United Kingdom Arm of the NU-AGE randomized controlled trial revealed that 1-year consumption of MedDiet along with Vitamin D by elderly subjects (65–79 years) showed MedDiet-dependent changes in T cell degranulation, cytokine production, and co-receptor expression. The elderly placebo group exhibited increased signs of IL-12 cytokine production, which might contribute to inflammaging. However, although not significant, the MedDiet supplemented group showed a declining tendency in IL-12 expression, indicating beneficial effects in elderly subjects. The authors suggested that prolonged MedDiet intervention studies are necessary to confirm the anti-inflammaging effects of MedDiet [180].

**Table 2:** List of selected nutrient compounds with clinical trials and human research targeting inflammaging.

Nutrient Compounds	Source	Study	Model (In Vitro and In Vivo)	Dose	Mechanism/s	Refs
Metformin	*Galega officinalis*	Cross sectional study	Human subjects	1000 mg/day/3 months	↑ autophagy and mitochondrial function	[160]
Zinc	Nutrient trace element	Randomized, double blinded placebo trial	Aged human subjects (56–83 years)	45 mg/d for 6 months)	↓ CRP, IL-6 and TNF-α levels	[166]
Gotu Kola	*Centella asiatica*	Single-blind, placebo-controlled clinical trial	Aged human subjects	2 mg/explant/ 5 days	↓ IL-1α	[169]
Whey protein	Dietary protein	Crossover designed randomized, acute clinical intervention study	Obese non-diabetic human subjects	45 g/12-week	↓ MCP-1 expression	[171]
Soy protein	Dietary protein	Randomized, double-blind, placebo-control, clinical trial	Healthy older women (>70 years.)	18 g/day/1 year	↓ IL-6 baseline	[174]
Rice NPN	Black rice	Randomized, double-blind, placebo-control, clinical trial	LPS induced macrophages. Aged subjects (65–75 years)	10 g dose/12 weeks	↓ TNF-α. Restored glucose, LDL and HDL levels	[178]
MedDiet	Mediterranean diet	Randomized controlled trial	Elderly subject (65–79 years)	MedDiet for 1 year	↓ IL-12 expression	[180]

Abbreviations: IL: Interleukin; LPS: Lipopolysaccharide; TNF-α: Tissue necrosis factor-alpha; HDL: high density lipoprotein; LDL: Low density lipoprotein; CRP: C-Reactive protein; Rice NPN: Rice natural peptide network; MCP-1: Monocyte chemoattractant protein-1, ↑: increased, ↓: decreased.

## 5. Patents Claims on Nutrient Compounds for Possible Anti-Inflammaging Effects

Till date, patents with direct mechanistic evidence on the role of nutrient natural compounds targeting inflammaging has not been published. However, we identified a few patent claims on nutrient compounds and natural product mixtures for their anti-aging effects based on anti-inflammatory properties. A list of selected patented nutrient compounds claimed in targeting inflammaging is shown in Table 3.

### 5.1. Cyclodextrins

**Figure 3:**
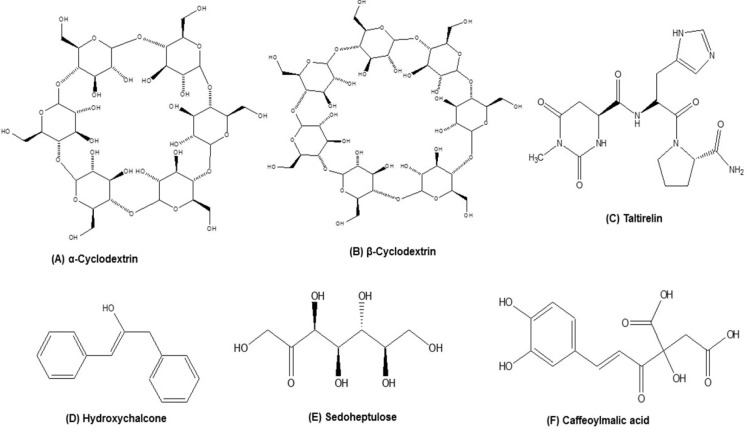
Chemical structures of selected patent agents targeting inflammaging.

In a recent patent by Asdera LLC, USA, the inventers claimed that cyclodextrin (α and β; Figure 3A,B) might be useful in the treatment and prevention of malignancies, in neurodegeneration (AD and PD), and other aspects of aging including the T2D and atherosclerosis [181]. Cyclodextrin is recognized as a safe food additive and its derivative 2-hydroxypropyl-p-cyclodextrin (HP-β-CD) has been reported to improve autophagy, which is a common factor in the etiology of ARDs [182]. In NSG mice infected with human MDA-MB-231 breast cancer cells, cyclodextrin (800 mg/kg) reduced the plasma cytokine levels such as IL-1β, IL-18, IL-6, and IL-8, significantly confirming that cyclodextrins reduce inflammation. It is well known that during inflammaging conditions there is an upregulation of inflammatory responses and downregulation of autophagy [183]. The inventors claim that cyclodextrins might be essential during early life in reducing inflammation, thereby improving autophagy. The authors concluded that cyclodextrins reduce chronic inflammation associated with aging.

### 5.2. Taltirelin

In a patent by Eolas Research LTD, Great Britain, the inventors claimed that a compound named taltirelin (N-[[(4S)-Hexahydro-l-methyl-2,6-dioxo-4-pyrimidinyl] carbonyl]-L-histidyl-L-prolinamide; Figure 3C) showed immense potential in the treatment of ARDs associated with cellular senescence, inflammaging, and autophagy [184]. It is well documented that the senescent cells develop SASP involving enhanced secretion of pro-inflammatory mediators contributing to inflammaging. Taltirelin, which is a thyrotropin-releasing hormone analogue, was well documented for its effects on improving neurological functions. The inventors indicated that taltirelin (10 mg/kg/day) treated to old fibroblast cells increased the expression of forkhead box protein (FOX) O1 and FOXO3 genes, which are regarded to be actively involved in various mechanisms including inflammaging, autophagy, and apoptosis. Taltirelin also increased the expression of SIRT1, which is known to be involved in several ARDs. Further, the EFNB1 gene is well documented to be involved in the adipose inflammatory response and in obesity. Taltirelin significantly increased the Enhrins (EFNB1) in old fibroblast cells compared with the control cells. Nrf2 is known to be involved in immunity and inflammation. During aging, there is a decrease in the Nrf2 activity. In this patent, the inventors claim that taltirelin significantly increased the Nrf2 activity in old fibroblasts compared with untreated controls.

### 5.3. Chalcones

Chalcones and their derivatives found in plants are well known to be used as medicaments and nutraceutical agents. In a patent application by III.XTII B.V, Netherlands, the inventors claimed that hydroxychalcone (33 µM; Figure 3D) exhibited a curative effect by reducing the blood IL-1β/Il-18 levels, inhibited NLRP3 inflammasome-mediated IL-18 expression in macrophages or dendritic cells, and inhibited caspase-1 in THP-1 macrophages or dendritic cells stimulated by LPS/ATP. In aging assay, hydroxychalcone treatment (50 µM) to N2, bristol (wild-type) strain nematodes increased the length of survival time of nematodes. The inventors concluded that hydroxychalcone possibly treats low grade inflammation and shows beneficial effects in targeting inflammaging and other ARDs [185].

### 5.4. Sedoheptulose

Sedoheptulose (Figure 3E), a monosaccharide with seven carbon atoms and a ketone functional group, is found abundantly in nature. It can be produced both naturally from fruits and vegetables, or synthetically. Sedoheptulose was pharmacologically reported to protect diabetic nephropathy by alleviating inflammatory response. In a patent applied by Medizinische Universität Wien 1090 Vienna, Austria, the inventors generated a transgenic mice overexpressing sedoheptulose kinase (CARKL) and tested various parameters including inflammatory markers, redox regulation, and physical activity by standard assays. The inventors claimed that sedoheptulose in the dose range of up to 150 mg/mL, decreased the LPS (7 mg/kg)-stimulated increase in inflammatory cytokines in endotoxemia in vivo murine model. The inventors indicated that sedoheptulose-administered animals showed suppressed inflammatory responses by reducing the inflammatory cytokine levels especially TNF-α or IL-6, or other inflammation markers when compared to wild type subjects. The inventors concluded that sedoheptulose might mitigate chronic low grade inflammation, thereby positively delaying inflammaging and controlling other ARDs [186].

### 5.5. Herbal Mixtures

In a patent by PharmacoGentetics Limited, Shatin, China, the inventors claimed that the natural herb mixtures including Radix *Bupleurum chinense*, Rhizoma *Corydalis yanhusuo*, Caulis *Polygonum multiflorum,* and Flos *Albizia julibrissin* delayed the aging process in D-gal-exposed mice. The inventors suggested that treatments helping in alleviating memory functions, attenuating oxidative stress, and reducing pro-inflammatory mediators might be crucial in preventing the accelerated aging seen in several ARDs. The inventors claimed that the mixture (120 mg/kg) treated for 2 months along with D-gal (150 mg/kg) significantly ameliorated the increased MDA (oxidative stress marker) and pro-inflammatory cytokines including TNF-α and IL-6 in the brain tissues of mice. The inventors concluded that the anti-inflammaging capacities of the claimed composition might be useful in preventing or delaying the inflammation-mediated aging-related disorders such as AD and PD (US 10,722,547 B2).

### 5.6. Nutrient Cosmeceutical Preparations

In another patent by LVMH Recherche GIE, France, the inventors claim that the aqueous extract of rose fruits (Evarant or Rose jardin de Granville variety) exhibited skin aging protective functions by ameliorating UV radiation-induced inflammaging of the skin. The inventors showed that rose fruit extract at 65 µg/mL concentration reduced the proinflammatory mediators such as IL-1α, IL-1β, IL-8, TNF-α, and NF-κB in keratinocyte cultures or co-cultures of sensory neurons and human keratinocytes induced by light stress (UV) chronic inflammation. The inventors indicated that the preventive effects of rose fruit extract on skin aging are linked with controlling inflammaging processes [187]. The same group also claimed that rose wood extract from similar species also exhibited potential anti-aging capacity on skin by preventing inflammaging [188].

In a study by a South Korean group of inventors from Seoul National University and Hyundai Bioland Co LTD, a cosmetic and functional food composition containing caffeoylmalic acid (Figure 3F) isolated from *Canavalia gladiata* reduced skin photo-aging. The inventors indicated that caffeoylmalic acid (20 µM) inhibited the UV radiation-induced increased expression of COX-2 in human keratinocyte HaCaT cells lines, one of the inflammaging targets [189]. Further, another South Korean company “COSMAX Inc” claimed that the cosmetic composition containing an extracts mixture of *Juglans nigra*, *Sophora japonica*, and *Pinus densiflora* exhibited potent anti-aging and anti-inflammatory effects by inhibiting the ROS and inflammatory mediators, thereby preventing skin aging mediated by UV-induced inflammation. The inventors concluded that equal portions of the three extracts significantly inhibited UV-induced IL-1β mRNA expression in human HS68 fibroblast cells, showing their efficacy for anti-aging and anti-inflammatory activities [190].

**Table 3:** List of selected nutrient compounds with patent claims targeting inflammaging.

Compound Name	Source	Model (In Vitro and In Vivo)	Dose	Mechanism/s	Refs
Cyclodextrin	Naturally occurring food additive	NSG mice infected with human MDA-MB-231 breast cancer cells	800 mg/kg once weekly for 6 weeks	↓ IL-1β, IL-18, IL-6, and IL-8. ↓ autophagy	[181]
Taltirelin	Thyrotropin-releasing hormone analogue	Old fibroblast cells	10 mg/kg/day	↑ Nrf2 activity	[184]
Chalcones	Plant derived nutrient	THP-1 macrophages	33 µM (in vitro)	↓ IL-1β/Il-18 levels	[185]
		N2, bristol (wild-type) strain nematodes	50 µM (in vivo)	↓ NLRP3 and ↓ caspase-1	
Sedoheptulose	Naturally from fruits and vegetables	LPS stimulated endotoxemia in vivo murine model and sedoheptulose kinase overexpressing mice	Up to 150 mg/mL	↓ TNF-α or IL-6	[186]

Abbreviations: LPS: Lipopolysaccharide; IL: Interleukin; TNF-α: Tissue necrosis factor-alpha; Nrf2: Nuclear factor erythroid-2-related factor 2; NLRP3: NOD-like receptor pyrin domain-containing protein 3, ↑: increased, ↓: decreased.

## 6. Conclusions and Future Perspectives

**Figure 4:**
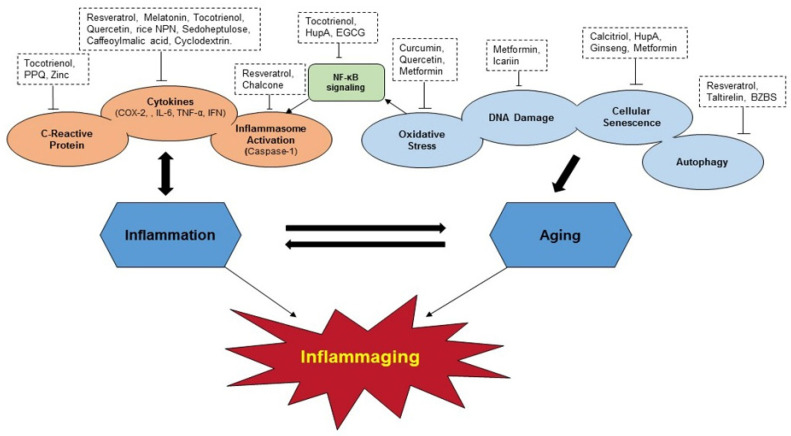
Schematic diagram on selected nutrient compounds and possible sites of action on inflammaging pathways. Inflammaging is caused by interactions at multiple levels of inflammatory and aging mechanisms. Aging-mediated activation involving oxidative stress, DNA damage, cellular senescence, autophagy, and parallel activation of inflammatory responses including increased CRP levels, pro-inflammatory cytokine levels (IL-6, IL-1, IL-1β, TNF-α, IFN, etc.), and inflammasome activation (caspase-1) are some of the key players known to involved in inflammaging and its propagation. Possible involvement of selected reviewed nutrient compounds was shown at various signaling steps. COX-2: Cyclooxygenase, IL: Interleukin, TNF-α: Tissue necrosis factor-alpha, IFN: Interferon, NF-κB: Necrosis Factor-Kappa B, PPQ: Pyrroloquinoline quinone, Rice NPN: Rice natural peptide network, HupA: Huperzine A, EGCG: Epigallocatechin-3-gallate, BZBS: BaZiBuShen.

Inflammaging is highly correspondent with ARDs. Although the manifestation of ARDs is not solely dependent on inflammaging, the pathomechanisms observed in ARDs can be correlated with inflammation. As aging is irreversible, the strategy to delay the aging process without favoring diseases could be the foremost. Thus, the idea of using natural nutrient compounds or nutraceuticals to reduce inflammaging can be very effective in controlling various geriatric syndromes and increasing the lifespan of aging populations to live healthier and longer. Over the past few decades, the literature has been suggesting that nutrients and food supplements from natural and synthetic sources hold promising agents in controlling the inflammatory conditions associated with aging and ARDs. In this review, we discussed several nutrient compounds that have proven to be promising in cellular and animal experimental models targeting inflammaging and increasing life span. Further, reports from clinical trials, human research, and patents claimed so far on selected nutrients with possible therapeutics on inflammaging and inflammaging-associated disorders was also discussed. A schematic diagram with selected nutrient compounds targeting inflammaging at various mechanistic pathways was shown (Figure 4).

Very few studies including the rice NPN, zinc supplementation, calcitriol, and MedDiet, have been investigated in clinical settings for their possible influence as potential anti-inflammaging agents. Further, the patents applied in recent years were focused primarily on anti-aging and anti-inflammatory effects in stimulated cells and animal tissues. A few other patents targeting inflammaging were mainly made from the cosmeceutical perspective, naming them as anti-aging or anti-photo aging agents by either preventing or delaying skin aging. Although the reported clinical studies and patent claims suggested to improve the over health conditions and life span by delaying the inflammaging processes, none of the agents were approved to be introduced in to the drug or nutraceutical market claiming therapeutic anti-inflammaging effects in aged populations associated with ARDs. Though targeting inflammaging by nutrient compounds is a prospective strategy to reduce the occurrence of ARDs and increase the lifespan of individuals, the direct link between inflammaging and ARDs has yet to be elucidated. To date, compounds targeting inflammation that can inhibit ARDs are believed to be the best choices against inflammaging. However, elucidating the specific pathomechanisms of respective diseases in relation to inflammaging could bring a phenomenal improvement. Moreover, the crucial question of whether there is a causal relationship between inflammaging and diseases needs to be resolved by performing extensive integrated biological and clinical research. Whether early modulation of inflammaging prevents or delays the onset of ARDs should be tested thoroughly by implementing stringent inflammaging experimental models. Since the verification of detailed inflammaging mechanisms in humans is still unclear, elaborated, well-controlled, and larger dose–response randomized clinical trials on the use of potential nutrient compounds in elderly populations and patients with ARDs are still necessary to identify threshold effects targeting inflammaging.
